# Complete Genome Sequence of *Photobacterium leiognathi* Strain JS01

**DOI:** 10.1128/genomeA.01396-17

**Published:** 2018-01-04

**Authors:** Justin Y. K. Soh, Colin W. Russell, Shannon N. Fenlon, Swaine L. Chen

**Affiliations:** a Infectious Disease Group, Genome Institute of Singapore, Singapore, Singapore; b Division of Infectious Diseases, Department of Medicine, Yong Loo Lin School of Medicine, National University of Singapore, Singapore, Singapore

## Abstract

*Photobacterium leiognathi* is a bioluminescent symbiont of fish of the *Leiognathidae* family. Here, we present the full-genome sequence of *P. leiognathi* strain JS01, a strain isolated from a nonluminescent *Loligo* sp. squid of Singaporean origin. No finished genome sequence of this species is currently publicly available.

## GENOME ANNOUNCEMENT

*Photobacterium leiognathi* is a Gram-negative bacterium that typically forms symbiotic relationships with fish of the *Leiognathidae* family (1). The bacteria colonize the photic organs of the fish, presumably receiving nutrients and oxygen in exchange for producing bioluminescence (2). The bioluminescence is thought to benefit the fish with respect to self-defense and avoiding predation (3, 4). *Photobacterium* species have also been found to associate with fish and tissues of decaying marine animals (1). We isolated *P. leiognathi* strain JS01 from a *Loligo* sp. squid purchased at a local wet supermarket in Singapore; of note, JS01 was luminescent on plates inoculated with the exterior of the ink sac, while other *Loligo* spp. (including the sample we purchased) are generally not.

*P. leiognathi* JS01 was grown on thiosulfate-citrate-bile salts sucrose agar. Colonies were scraped from plates for genomic DNA purification using the Qiagen QiAamp DNA minikit. A sequencing library was prepared from 1 µg of unsheared genomic DNA using the Oxford nanopore ligation sequencing kit 1D R9 version (SQK-LSK108). This library was sequenced using 1D nanopore sequencing by ligation using the Oxford Nanopore MinION MKII DNA sequencer and the FLO-MIN106 R9 with Spot-ON. A total of 44,729 reads were obtained from a 25-hour sequencing run. A total of 449,533,611 bp was collected. The mean read length was 10,050 bp, and the longest read was 273,911 bp. The reads were corrected and assembled using Canu (version 1.3), specifying only the “-nanopore-raw” and “genomeSize=5m” parameters. The assembly was further polished using nanopolish (version 0.8.3), specifying only the “--min-candidate-frequency 0.1” and “--max-haplotypes 1000000” parameters, and then manually trimmed to remove duplicated sequence present due to a circular topology. The JS01 assembly consists of three unitigs of 3,251,164 bp, 1,563,892 bp, and 59,473 bp. The G+C content is 41.2%. Annotation of the draft assembly was performed using the NCBI Prokaryotic Genome Annotation Pipeline (PGAAP), which predicted 2,606 protein-coding sequences, 57 rRNA (2 incomplete), and 205 tRNA genes; however, 1,682 pseudogenes were also predicted (1,642 frameshifted), which was likely caused by insertion and deletion errors that are common with Oxford Nanopore Technologies (ONT) sequencing. Other *Photobacterium* species (*Photobacterium damselae*,* P. profundum*,* and P. gaetbulicola*) typically have two chromosomes of 3.13 to 4.09 Mbp and 1.05 to 2.24 Mbp, as well as a variable number of plasmids ranging from 50 to 110 kbp, with a high number (29 to 49 rRNAs and 131 to 194 tRNAs) of RNA genes (5–7). We therefore suspect that our assembly of *P. leiognathi* is nearly complete, with the two larger unitigs representing the two chromosomes and the smaller 59-kb unitig a plasmid, but we have not verified this.

### Accession number(s).

The complete genome sequence of *P. leiognathi* JS01 was deposited in GenBank under the accession no. PDNH00000000. The version described in this paper is version PDNH02000000. The assembly and raw reads are collected together under BioProject PRJNA413640.
